# Take it to the limit: The limitations of energetic explanations for birth timing in humans

**DOI:** 10.1093/emph/eoad035

**Published:** 2023-10-31

**Authors:** Cédric Cordey, Nicole M Webb, Martin Haeusler

**Affiliations:** Institute of Evolutionary Medicine, University of Zürich, Winterthurerstrasse 190, 8057 Zürich, Switzerland; Institute of Evolutionary Medicine, University of Zürich, Winterthurerstrasse 190, 8057 Zürich, Switzerland; Department of Palaeoanthropology, Senckenberg Gesellschaft für Naturforschung, Senckenberganlage 25, 60325, Frankfurt am Main, Germany; Institute of Evolutionary Medicine, University of Zürich, Winterthurerstrasse 190, 8057 Zürich, Switzerland

**Keywords:** obstetrical dilemma, pregnancy, energetics, birth, EGG hypothesis, secondary altriciality

## Abstract

A hallmark of modern humans is that our newborns are neurologically immature compared to other primates. It is disputed whether this so-called secondary altriciality evolved due to remodelling of the pelvis associated with bipedal locomotion, as suggested by the obstetrical dilemma hypothesis, or from maternal energetic limitations during pregnancy. Specifically, the ‘Energetics of Gestation and Growth’ (EGG) hypothesis posits that birth is initiated when foetal energy requirements exceed the maximum sustained maternal metabolic rate during pregnancy at around 2.1 × basal metabolic rate (BMR) of the non-pregnant, non-lactating condition (NPNL). However, the metabolic threshold argued under the EGG framework is derived from one study with a small sample size of only 12 women from the UK. Accordingly, we performed a meta-analysis of all published studies on metabolic scopes during pregnancy to better account for variability. After excluding 3 studies with methodological issues, a total of 12 studies with 303 women from 5 high- and 3 low-income countries were analysed. On average, pregnancy was found to be less metabolically challenging than previously suggested. The studies revealed substantial variation in metabolic scope during pregnancy, which was not reflected by variation in birth timing. Further, in a third of the studies, the metabolic rates exceeded 2.1 × BMR_NPNL_. Our simulation of foetal energy requirements demonstrated that this metabolic threshold of 2.1 × BMR_NPNL_ cannot realistically be crossed by the foetus around the time of birth. These findings imply that metabolic constraints are not the main limiting factor dictating gestation length.

## INTRODUCTION

Newborn modern humans weigh approximately twice as much as those of great apes, both absolutely and relative to adult body mass, yet neonatal brain size is only 28% of the mother’s brain size, whereas it averages 43% in non-human primates [1–3]. Human newborns are, therefore, remarkably neurologically immature and helpless at birth. This condition has been labelled ‘secondary altriciality’, in contrast to the precociality of other primates, but also to distinguish it from true altriciality, the primitive condition for mammals [4]. However, whether secondary altriciality is due to pelvic constraints [5] or maternal metabolic limitations [6] remains unclear. The former hypothesis attributes secondary altriciality to an evolutionary trade-off between natural selection for a sufficiently large birth canal in females and a biomechanically efficient pelvis with a short sacroiliac-to-hip joint distance adapted for bipedal locomotion in both sexes [7–10]. While the roots of this hypothesis go back to the beginning of the 20th century [10], it was Washburn [7] who aptly named it the ‘obstetrical dilemma’.

The obstetrical dilemma hypothesis has recently been criticized from various perspectives. Most of these critiques focus only on single aspects like the energetic consequences of pelvic width [11] or factors influencing pelvic width variation other than obstetrical selection pressures [12–17]. However, since Washburn [7] emphasized the evolutionary shortening of the hipbone rather than pelvic width, these criticisms miss the very foundation of the obstetrical dilemma in its original form (see also the reviews in Refs. [10, 18]).

**Figure 1.**
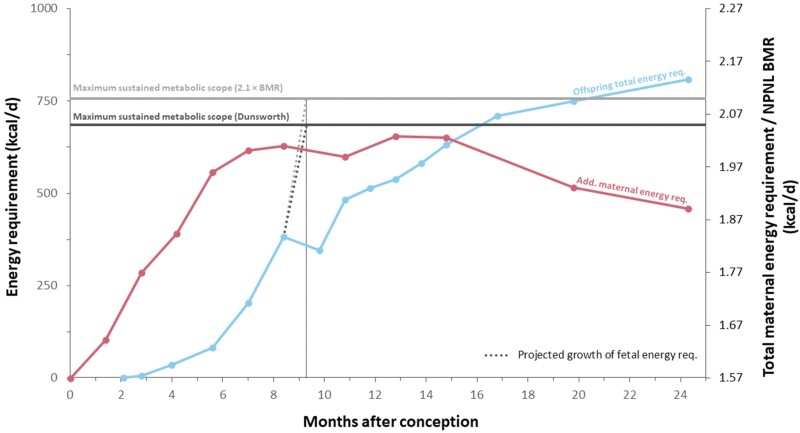
Comparison of offspring energy requirements to the additional maternal energy requirements during pregnancy and lactation (see Dunsworth *et al.* [6]). The additional maternal energy requirements are based on the study of Goldberg *et al.* [24] and seem to plateau in the third trimester, thus approaching a theoretical maximum sustained metabolic scope, which was suggested to lie between 2.04 × BMR (inferred from Fig. 3 in Dunsworth *et al.* [6]) and 2.1 × BMR. According to Dunsworth *et al.* [6], this maternal threshold is crossed by the supposedly exponentially increasing offspring energy requirements at the time of birth, thus inducing labour

The most comprehensive critique of the obstetrical dilemma to date is offered by the ‘Energetics of Gestation and Growth (EGG) hypothesis’. It not only questions that difficult birth was caused by pelvic adaptations to bipedal locomotion but also provides an alternative explanation for secondary altriciality, thus countering two of the central pillars of the obstetrical dilemma hypothesis [6, 19]. In analogy to the constrained model of total energy expenditure (TEE) for physical activity [20–23], the EGG hypothesis posits that the increasing energy demands of the pregnant mother approach a plateau towards the end of the third trimester, suggesting the presence of a ‘metabolic ceiling’ or threshold. The energy requirements of the foetus are said to simultaneously increase in an exponential manner, and when they cross the mother’s maximum sustained metabolic rate, birth would be initiated (Fig. 1). Yet, the EGG hypothesis also lacks an explanation for the high prevalence of birth difficulties and the marked degree of pelvic sexual dimorphism typical of all modern human populations.

The EGG hypothesis is technically an extension of Ellison’s [25] ‘metabolic cross-over’ hypothesis, which suggests that towards the end of pregnancy, maternal metabolic capacity is insufficient to maintain the energy supply through the placenta for the growing foetal brain and to ensure the foetus can acquire sufficient fat reserves to survive the postnatal period. Particularly, the placental transfer of fatty acids was thought to constitute a bottleneck towards the end of pregnancy. The resulting starvation of the foetus would lead to a surge of foetal cortisol that triggers uterus contractions and thus initiates labour. Ellison [25] accordingly argued that lactation is far more efficient in supplying the growing infant with nutrients than the placenta. In fact, the energetic demands of lactation are notably higher than the energetic demands of pregnancy [6, 26]. Moreover, lactation allows for a more efficient transfer of glucose to the growing foetal brain [22].

However, since the formulation of the metabolic cross-over hypothesis in 2001, *in vitro* and *in vivo* studies of the placental transport of ^14^C-labelled fatty acids showed that this process occurs rapidly [27, 28]. It, therefore, does not seem to constrain the availability of nutrients to the foetus [10]. Support for the efficiency of the human placenta also comes from the observation that the maternal metabolism can supply twins, multiples and, in exceptional cases, up to 10 kg heavy foetuses without negative long-term outcomes [29]. Moreover, the endocrine clock seems to work differently in humans than in other mammals like sheep, on which Ellison’s [25] metabolic crossover hypothesis, and by extension the EGG hypothesis, are based. Thus, in contrast to the sheep model, humans do not show a dramatic surge of cortisol at the end of pregnancy [30]. Nevertheless, mean maternal cortisol levels are more than 10 times higher than foetal values, and a significant percentage of the maternal cortisol passes through the placenta [31], so that the increasing cortisol levels in the foetal circulation cannot readily be attributed to starvation as suggested by Ellison’s [25] metabolic cross-over hypothesis.

The maternal energetic threshold of the EGG hypothesis, the so-called ‘metabolic ceiling’, was proposed to lie at approximately 2.0–2.1 times the basal metabolic rate of the non-pregnant, non-lactating condition (BMR_NPNL_), which only slightly exceeds the metabolic scope during lactation [6]. Although many authors measured energy expenditure during pregnancy in humans (see reviews in Ref. [26, 32]), the EGG hypothesis is based on the metabolic data of a single study by Goldberg *et al.* [24] with only 12 women before and during pregnancy from a socioeconomically strong, affluent society. Moreover, a recent extrapolation from ultralong athletic endurance activities suggests a maximum sustained metabolic rate of 2.5 × BMR_NPNL_ for a 280-day-long event like pregnancy [33]. Therefore, the metabolic ceiling of pregnant women could be significantly higher than the 2.0–2.1 × BMR_NPNL_ threshold assumed by the EGG hypothesis. However, the energetic requirements of the foetus would need to increase enormously to cross those of the expectant mother at the time of birth if this threshold would be significantly higher than 2.0 to 2.1 × BMR_NPNL_. It is, therefore, unclear whether metabolic limitations are the sole determinant for gestation length in humans as suggested by the EGG hypothesis.

Recently, Prado-Novóa *et al.* [34] also demonstrated that pregnant women expend less energy than non-pregnant women of the same body weight due to their higher percentage of fat mass, which is more passive in terms of metabolic energy requirements. As pregnant women had the same relationship between body mass and BMR, and between body mass and the cost of locomotion, the authors concluded that pregnancy could not be as constrained as previously suggested [34].

Given these issues surrounding the EGG hypothesis, the present study aims to expand the data on which the hypothesis of Dunsworth *et al.* [6] rests and to more accurately approximate the point at which a metabolic ceiling is reached during pregnancy, if at all since these both determine whether it can realistically be crossed by the energetic demands of the growing foetus. Because foetal energy requirements are mainly determined by foetal body mass and cost of growth [6], the present study also explores how the birth weight would be affected by different maximum sustained maternal metabolic scopes during pregnancy. Moreover, we examine whether these studies indicate that gestation length correlates with sustained maternal metabolic scope as predicted by the EGG hypothesis. Collectively, this meta-analysis attempts to test the validity of the assumption that maternal energetics is the main determinant governing gestation length.

## METHODOLOGY

### Study selection

We identified 15 studies reviewed by Butte and King [26] and Savard *et al.* [32] that measured total energy expenditure during pregnancy in humans. Three of the studies did not measure TEE in the non-pregnant, non-lactating condition or estimated TEE using different body measurements and qualitative assessments of activity level and were consequently excluded. Specifically, the study of Lawrence and Whitehead [35] was omitted because it did not report BMR nor any other characteristics before or during pregnancy and only provided TEE for weeks <9 and weeks 28–40 of pregnancy. The study of Most *et al.* [36] was excluded because only obese women were studied and non-pregnant, non-lactating values were missing for all relevant measurements. Estimating these non-pregnant, non-lactating values in obese women is not trivial, since BMR changes differently in lean and obese women in the early weeks of pregnancy [37, 38]. Finally, the study of Poppitt *et al.* [39] was excluded because they measured 24h-energy expenditure of Gambian women during pregnancy using a metabolic chamber in which the participants performed controlled activities that resembled a sedentary day. This experimental setup potentially differed from a typical day of these Gambian women. In fact, using two-tailed *t*-tests, we found the mean 24 h-energy expenditure observed by Poppitt *et al.* (1545 kcal/d) [39] was significantly lower than the free-living energy expenditure of Gambian women reported by both Singh *et al.* (2309 kcal/d; *P* < 0.05) [40] and Heini *et al.* (2072 kcal/d, *P* < 0.05) [41], although all three studies observed a similar non-pregnant, non-lactating BMR (mean 1234 kcal/d, SD 33 kcal/d).

This left 12 studies that were included in the present meta-analysis (Table 1). Some of them examined different subgroups of varying sample sizes. For example, Forsum *et al.* [43] studied 28 women before and during pregnancy, of whom 22 completed the metabolic measurements up until week 30, while only 19 women remained until week 36. Conversely, Butte *et al.* [49] measured metabolic rates in women with low, normal and high BMI, of which the weighted means of the measured metabolic rates were included in the present analysis. Weighted means were also calculated for Panter-Brick [44] who compared TEE in pregnancy for the four different seasons of the year. Two studies, that is, Butte *et al.* [46], performed in the USA, and Abeysekera *et al.* [50], performed in Australia, did not measure BMR or TEE for the non-pregnant, non-lactating condition. For further analysis, we, therefore, added to these two datasets the weighted mean of the non-pregnant, non-lactating TEE and BMR of all other studies performed in socioeconomically stronger countries [24, 42, 43, 45, 48, 49]; socioeconomically stronger countries are defined according to their gross domestic product [51] and the Human Development Index [52]. Specifically, in the present they include studies of the Netherlands, Sweden, the UK, Australia, and the USA.

**Table 1.** Energy requirements for human mother during pregnancy (calculated using the same assumptions as Dunsworth *et al.* [6])

Reference	Country	Measurement method	n	Week of gestation	Months postpartum	Weight (kg)	TEE (kcal/d)	Fat deposition (kcal/d)^a^	Protein deposition (kcal/d)^a^	Additional energy req. (kcal/d)^b^	Total energy req. (kcal/d)^c^	BMR (kcal/d)	Metabolic scope^d^
Goldberg *et al.* [24]	UK	DLW	12	NPNL		61.7	2275				2275	1446	1.57
(used by Dunsworth *et al.* [6])				6		62.2	2323	55	0	103	2379	1503	1.64
				12		63.3	2428	128	4	285	2561	1489	1.77
				18		65.4	2457	201	8	391	2666	1494	1.84
				24		68.7	2622	191	21	558	2834	1580	1.96
				30		71.7	2677	181	33	616	2891	1649	2.00
				36		73.6	2689	181	33	628	2903	1804	2.01
Singh *et al.* [40]	Gambia	DLW	6	NPNL		50	2072				2072	1197	1.73
				20		60	2581	201	8	718	2791	1427	2.33
Goldberg *et al.* [42]	UK	DLW	10	NPNL			2337				2337	1401	1.67
				36			2469	180	33	345	2682	1742	1.92
Heini *et al.* [41]	Gambia	DLW	7	NPNL		50	2309				2309	1262	1.83
			8	12		54	2522	55	0	268	2577	1346	2.04
			8	24		55	2161	201	8	61	2370	1312	1.88
			9	36		65	2278	180	33	182	2491	1632	1.97
Forsum *et al.* [43]	Sweden		22	NPNL		61.0	2486				2486	1338	1.86
				17		63.7	2294	201	8	18	2504	1434	1.87
				36		70.2	2988	180	33	715	3201	1649	2.39
Panter-Brick *et al.* [44]	Nepal	Indirect calorimetry + activity diaries	19	NPNL		47	2382				2382	1242	1.92
			24	36		53	2231	180	33	61	2444	1275	1.97
de Groot *et al.* [45]	Netherlands	Room calorimetry	12	NPNL		61.4	2063				2063	1391	1.48
				12		62.1	2087	55	0	79	2142	1499	1.54
				23		66.4	2170	201	8	317	2380	1558	1.71
				34		72.3	2376	180	33	526	2589	1728	1.86
Butte *et al.* [46]	USA	Room calorimetry	67	NPNL							2405^e^	1335^e^	1.76
				37			2266	180	33		2479		1.83
Dufour *et al.*	Colombia	Indirect calorimetry + heart rate method	114	NPNL		50	2435				2435	1248	1.95
			40	14		54	2414	55	0	34	2469	1274	1.98
			54	25		55	2223	201	8	-3	2432	1326	1.95
			43	35		65	2400	180	33	177	2613	1415	2.09
Kopp-Hoolihan *et al.* [48]	USA	DLW	10	NPNL			2206				2206	1315	1.68
				9			2048	55	0	-102	2104	1305	1.60
				24			2412	128	21	355	2561	1544	1.95
				36			2729	180	33	737	2943	1804	2.24
Butte *et al.* [49]	USA	DLW + room calorimetry	63	NPNL		59	2480				2480	1304	1.90
				9		60						1344	
				22		65	2481	201	8	210	2690	1425	2.06
				36		72	2645	180	33	378	2858	1649	2.19
Abeysekera *et al.* [50]	Australia	Accelerometer	26	NPNL							2405^e^	1335^e^	1.80
				12		62	2274	55	0	-40	2329	1499	1.72
				24		66	2447	201	8	287	2657	1558	1.96
				34		72	2453	180	33	297	2666	1728	1.97

NPNL, non-pregnant, non-lactating; TEE, total energy expenditure; BMR, basal metabolic rate; DLW, doubly labelled water method; req., requirements.

^a^Required energy for fat/protein accretion (tissue gain) during pregnancy (Table 8 in Butte and King [26]) .

^b^Required energy above NPNL value, including increment in TEE and tissue gain during pregnancy.

^c^TEE + tissue gain.

^d^Total energy req./NPNL BMR.

^e^Weighted mean calculated using the data of studies performed in the USA, the UK, the Netherlands and Sweden.

### Sustained metabolic scope during pregnancy

Energy expenditure generally shows physiological limits during prolonged physical activities [33, 53, 54]. To normalize for body size and differences in basal energy expenditure, the maximum sustained metabolic scope (SusMS_max_) is defined as the ratio of TEE (kcal/d) that can be maintained without depleting energy reserves, thus preserving constant body mass, and basal metabolic rate (BMR, kcal/d) [33, 53, 54]. To estimate maximum sustained metabolic rate, that is, the alleged metabolic ceiling during pregnancy, we followed Dunsworth *et al.* [6] in adding the energy requirement that accounts for gestational tissue gain to TEE. For this, the energy cost of increments in total body protein (*E*_P_) and fat mass (*E*_F_) during pregnancy was taken from Table 8 of Butte and King [26]. To obtain the metabolic scope during pregnancy, total energy requirement (TEE + additional energy requirement) was divided by the non-pregnant, non-lactating BMR. The maximum sustained metabolic scope is thus calculated as


$${\text{SusMS}}_{\text{max pregnancy}}=\;\frac{\left(\text{TEE}+E_{\text{P}}+E_{\text{F}}\right)}{{\text{BMR}}_{\text{NPNL}}}=\;\frac{\text{Metabolic ceiling}}{{\text{BMR}}_{\text{NPNL}}}.$$

### Offspring energy requirement and birth weight estimation

For all studies, the same offspring energy requirements were used as by Dunsworth *et al.* [6] (see their Supplementary Table S2). These values were subsequently used to estimate the foetal weight in pregnancy week 40 for different foetal energy requirements based on an average foetal weight of 2.97 kg in pregnancy week 36 (Table 2).

**Table 2.** Birth weight estimation based on foetal energy requirement under the assumption of the EGG hypothesis that labour is initiated when foetal energy requirements exceed the maximum sustained maternal metabolic scope

Maximum sustained metabolic scope^a^	1.8	1.9	2.1	2.3	2.5
Foetal TEE (kcal/d)^b^	218	257	354	447	544
Energy for growth (kcal/d)^c^	76	170	395	600	805
Foetal energy requirement (kcal/d)^d^	294	427	749	1050	1350
Predicted birth weight (kg)^e^	3.34	3.8	4.9	5.9	6.9

^a^Multiple of the maternal basal metabolic rate of the non-pregnant, non-lactating condition after Dunsworth *et al* [6].

^b^TEE—total energy expenditure.

^c^Based on a foetal weight of 2.97 kg in pregnancy week 36 (Supplementary Table S2 in Dunsworth *et al.* [6]).

^d^Sum of foetal TEE and energy for growth.

^e^Gestation length assumed to be 40 weeks.

## RESULTS AND DISCUSSION

**Figure 2.**
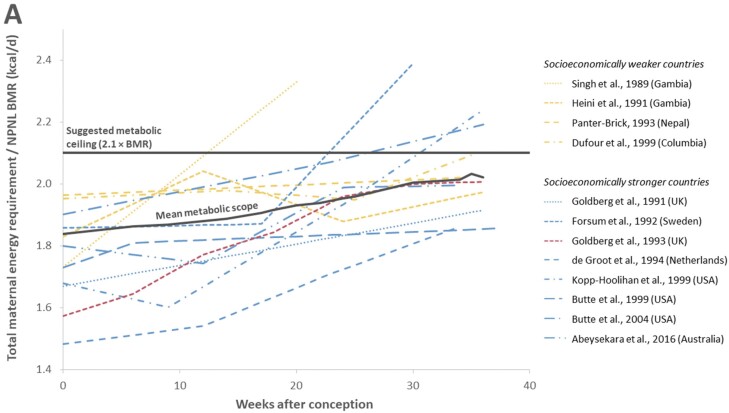

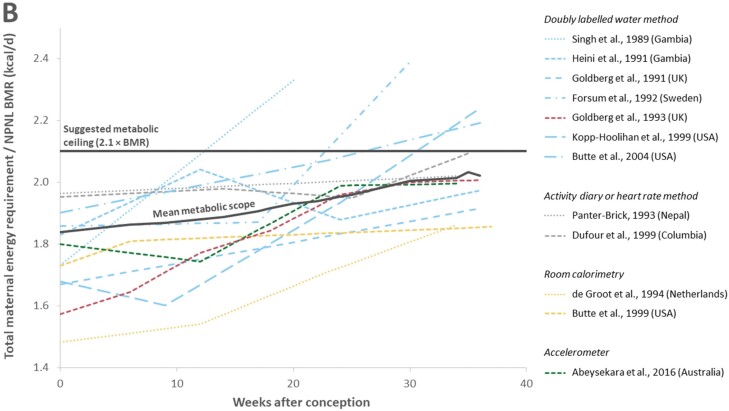
(A) Comparison of the sustained metabolic scope during pregnancy across the different studies. On average, it shows a steady increase until birth (black line) rather than a plateau as suggested by the Goldberg *et al.* [24] study (red) that was used by Dunsworth *et al.* [6] to establish the EGG hypothesis and to infer a metabolic ceiling for pregnancy at about 2.1 × BMR. The variation in metabolic scopes among all studies is considerable, with women from socioeconomically weaker countries (yellow) generally showing a higher sustained metabolic scope than women from socioeconomically stronger countries (blue), and 4 of the 12studies exceed the threshold of 2.1 × BMR before pregnancy week 36. (B) Comparison of the different measurement methods used by the included studies. Goldberg *et al.* [24] and six other studies used the doubly labelled water method (blue). Two studies used the heart rate and activity diary methods, respectively (grey), two other studies indirect or room calorimetry (yellow), and one study relied on accelerometers (green). Differences in sustained metabolic scope seem to be caused by behavioural and cultural differences rather than by the measurement method (For interpretation of the references to colour in this figure legend, the reader is referred to the web version of this article.)

The maternal energy requirements during pregnancy calculated for the included 12 studies using the same approach as in Dunsworth *et al.* [6] are shown in Fig. 2 and Table 1. In this figure, three aspects are particularly noteworthy: (i) The calculated sustained metabolic scope during pregnancy showed considerable variation among the 12 studies, with one third of the studies surpassing the metabolic ceiling suggested by the EGG hypothesis of 2.1 × BMR. (ii) The mean maternal metabolic scope of the included 12 studies increased less steeply than in the Goldberg *et al.* [24] study that served as the basis of the EGG hypothesis. On average, pregnancy seemed to be less energetically costly than Dunsworth *et al.* [6] proposed based on the Goldberg *et al.* [24] study. (iii) The mean maternal metabolic scope increased steadily without plateauing towards the end of pregnancy. Besides the study of Goldberg *et al.* [24], only two other studies indicated evidence of an anticipated plateau of maternal metabolic scope during pregnancy [41, 50], although in one of them [41] the maximum maternal metabolic scope was already reached in week 10. This pattern is, therefore, not suggestive of an energetic ceiling approached by the women shortly before birth.

### Variation in TEE and metabolic scope

Among the different studies, non-pregnant, non-lactating TEE ranged from 2063 kcal/d to 2480 kcal/d and the metabolic scope from 1.48 to 1.95. During pregnancy, the maximum maternal energy requirements ranged from 2444 kcal/d to 3201 kcal/d, which corresponds to metabolic scopes between 1.86 and 2.39. This might partly reflect the inter-individual variation in fat and protein deposition during the late stages of pregnancy. However, the reviewed studies did not always measure energy expenditure at the same timepoints, which impedes direct comparison. In some studies, the additional energy requirement merely reached 100 kcal/d, while in others over 700 kcal/d were expended more on average.

In 4 of the 12 studies [40, 43, 48, 49], the mean metabolic rate exceeded 2.1 × BMR_NPNL_ before or by pregnancy week 36. Noteworthy is particularly the metabolic rate of the Gambian women studied by Singh *et al.* [40] that equalled 2.33 × BMR already around pregnancy week 20 (SD = 5), but the sample size was low (*n* = 6) and no later measurements were provided. The authors indicated, however, that the non-pregnant, non-lactating women and the pregnant women were not the same individuals. In Butte *et al.* [49] (USA; *n* = 63), not only the weighted mean metabolic rate of the three subsamples (2.19 × BMR_NPNL_ in pregnancy week 36) exceeded the threshold of 2.1 × BMR but also each subsample itself (2.21 × BMR_NPNL_ in the low BMI group; 2.20 × BMR_NPNL_ in the normal BMI group and 2.15 × BMR_NPNL_ in the high BMI group). Finally, a high mean metabolic rate was also reported by Dufour *et al.* [47] for a study performed in Columbia, being at 2.09 × BMR_NPNL_ in pregnancy week 35, but no data have been recorded for week 36 or later.

### Socioeconomic status

Pregnant women potentially use different strategies to lower their own energy expenditure. Such strategies could include energy-sparing mechanisms, like the reduction of the maternal BMR [39], or lowering the physical activity level [26]. However, certain strategies may differ in their effectiveness depending on lifestyle and behavioural preferences.

Differences in the socioeconomic background of the study populations could explain part of the variation as the mean metabolic scope, particularly before pregnancy, was generally higher in socioeconomically weaker countries. Thus, socioeconomic background has been linked to physically more demanding workloads [55]. Additionally, the effects of poverty on workload and consequently physical activity during pregnancy have been considered [26, 56]. On the other hand, the ability to reduce physical activity during pregnancy may be a modern phenomenon. For instance, pregnant animals in the wild cannot reduce their activity owing to environmental pressures including predation [57], and it can, therefore, be assumed that early hominins faced similar pressures.

### Measurement methods

It is possible that the measurement methods contributed to the high variation of the metabolic rates (Fig. 2B). Thus, the two studies based on indirect calorimetry [45, 46] stand out by their relatively low maternal metabolic scopes: de Groot *et al.* [45] observed the overall lowest metabolic scopes, and Butte *et al.* [46] found the lowest metabolic scopes towards the end of pregnancy. Indirect calorimetry measures O_2_ consumption and CO_2_ production and is considered the most accurate method to measure energy expenditure in a clinical setting [58]. It is therefore mostly used to determine only BMR [41, 42, 45–47, 49]. In both studies [45, 46], the maternal metabolic scope increased in a similar pattern to the overall mean. This is in line with other results showing that indirect calorimetry yields comparable results to the doubly labelled water or flex heart rate methods [59]. We consequently suggest that other aspects of human pregnancy such as physical activity levels likely contribute more to the high variability observed in sustained metabolic scope and additional maternal energy requirements (see also Ref. [25]), rather than the measurement methods used in the reviewed studies.

**Figure 3.**
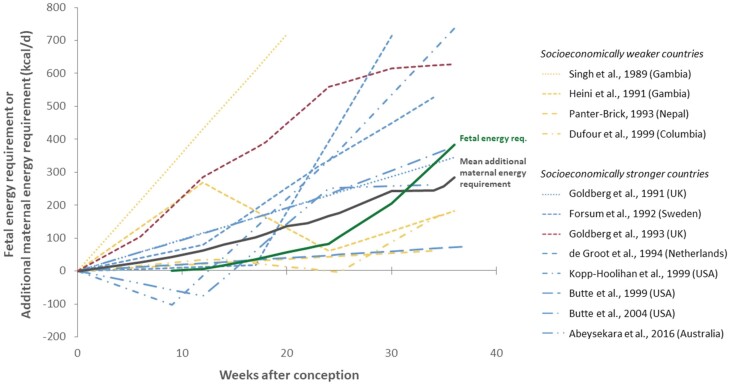
Comparison of foetal energy requirement (according to Dunsworth *et al.* [6]) with the additional maternal energy requirement of the studied women during pregnancy across the included studies. Towards the end of pregnancy, in 6 of the 12 included studies the additional energy requirement is lower than the foetal energy requirement

In the free-living context, the doubly labelled water method (DLW) is usually considered the gold standard, as it imposes a minimal burden to the participants and is sufficiently accurate, having a precision of 2–8% compared against respiratory gas exchange [60–62]. The DLW method was used by seven studies in the present meta-analysis to measure TEE, and the thus assessed metabolic scopes ranging from the second lowest to the highest values (see Fig. 3).

Another method to measure TEE during pregnancy is the flex heart rate method, which was used by Dufour *et al.* [47] in Columbian women. This method yielded similar results to indirect calorimetry and DLW [59, 63]. On the other hand, Dufour *et al.* [47] assessed BMR_NPNL_ with indirect calorimetry. Panter-Brick [44] relied on an alternative method to assess TEE during pregnancy in Nepali women, which was based on activity diaries. The data for the activities were derived from indirect calorimetry measurements, while factors were used for the unaccounted time during the day. BMR_NPNL_ was estimated by using an age-specific predictive equation based on individual height and month-specific mass [60]. The studies of Dufour *et al.* [47] and Panter-Brick [44] showed the highest non-pregnant, non-lactating metabolic scopes, which might be due to the high levels of physical activities observed in these women, while the metabolic scopes at the end of pregnancy were close to the average of all studies.

Finally, Abeysekera *et al.* [50] based their analysis on accelerometer data (SenseWear® Armband) that generally show a high correlation with indirect calorimetry (*r* = 0.87) [64]. Nevertheless, it is possible that we overestimated the metabolic scope during pregnancy of this study performed in Australia since we approximated the non-pregnant, non-lactating BMR by the mean of studies performed in the USA, the UK, the Netherlands and Sweden. This approximation might be too low considering a hypothetical BMR increase of 569 kcal/d and a drop in metabolic scope within the first trimester (see Table 1).

### Pattern of the additional maternal energy requirement

An examination of the additional energy required during pregnancy revealed on average a steady increase during the entire course of pregnancy rather than a plateau during the third trimester as suggested by Dunsworth *et al.* [6], which was inferred from the Goldberg *et al.* [24] study (Fig. 3). Some studies showed, however, only a minimal increase of about 70 kcal/d until the end of the third trimester. In four studies, the additional maternal energy requirements were lower than the foetal energy requirements for a large part of the pregnancy [41, 44, 46, 47]. However, the additional maternal energy requirement observed by de Groot *et al.* [45] showed a similar pattern as the overall mean, suggesting that indirect calorimetry for the measurements of TEE did not *a priori* yield different results compared to DLW.

Moreover, the women studied by Abeysekera *et al.* [50] showed a drop in the additional maternal energy requirements at around the 24th week of pregnancy. Similar declines of the additional maternal energy requirements were observed by Heini *et al.* [41] and Dufour *et al.* [47] around the 25th week of gestation. This decline in sustained metabolic scope observed in 4 of the 12 studies might be related to the reduction of physical activity that has often been described towards the end of pregnancy [50, 65–67], and multiple factors, like cardiovascular or respiratory changes, weight gain and low back and pelvic pain, may also account for it [68, 69]. It, therefore, seems to be too simplistic to attribute the lower physical activity level towards the end of pregnancy solely to a potential energetic cap, especially since the pattern is not consistent across all studies.

### Birth timing

If metabolic limitations were the sole determinant of birth timing, not only women with high sustained metabolic scopes at the end of pregnancy would be expected to show relatively shorter gestation lengths but also the opposite would be expected for women with relatively low sustained metabolic scopes. Butte *et al.* [31] reported that five women had been excluded from the study because of preterm births, and two additional women miscarried. However, neither the causes nor the week of the preterm deliveries and miscarriages were explicitly stated. In Goldberg *et al.* [24], 3 out of the participating 33 women miscarried before pregnancy week 16, and only 12 completed the study, although no preterm deliveries were reported. De Groot *et al.* [45] reported one post term delivery with a gestation length of 296 days out of 12 births, which falls within the prevalence of post-term births in Europe (0.4–8%) [70]. The 12 women of de Groot *et al.* [45] showed overall the lowest metabolic scope during pregnancy in comparison to the other studies (1.86 × BMR_NPNL_ for gestation week 34; Fig. 2). The same metabolic rate was reported by Butte *et al.* [46] for pregnancy weeks 35–36. However, all women delivered at full term at a mean gestational age of 39.1 weeks (SD = 1.3), and it seems unlikely that these women could have surpassed the presumed metabolic ceiling of 2.1 × BMR_NPNL_ at the time of birth (see Fig. 2). In fact, a value of approximately 750 kcal/d of additional maternal energy requirement and a sustained maternal metabolic rate of 2.1 × BMR_NPNL_ were only observed about 5 months after childbirth by de Groot *et al.* [45], yet this included the energy demands for breastfeeding. Similarly, no preterm births were reported by Abeysekera *et al.* [50], although in this study, the additional maternal energy requirements dropped below the foetal energy requirements at around the 34th week of pregnancy.

### The level of the ‘metabolic ceiling’ and foetal energy requirements

Another question is how high this argued metabolic ceiling is during pregnancy. Four of the 12 analysed studies were found to exceed a 2.1 × BMR_NPNL_ threshold several weeks before birth. Nevertheless, no preterm births were reported by these studies. This is supported by numerous studies that found no association between low or high physical activity levels during pregnancy and preterm delivery [71–73]. Yet, this suggests that the metabolic ceiling might be higher than proposed by the EGG hypothesis [6], and perhaps as high as 2.5 × BMR_NPNL_, which was inferred for pregnancy from an extrapolation of ultralong athletic endurance activities [33].

The mean birth weight reported in the present studies was 3.35 ± 0.44 kg. A foetus of that size requires approximately 300 kcal/d, if it grows from an average weight of 2.97 kg in pregnancy week 36 (Supplementary Table S2 in Dunsworth *et al.* [6]). However, if the maternal metabolic ceiling would be higher, this would also imply higher foetal energy demands. This is because the EGG hypothesis assumes that birth is initiated when the metabolic ceiling of the mother is surpassed by the energetic requirement of the foetus [6]. Specifically, since the energy demands of the foetus are mainly determined by foetal body mass and cost of growth, the hypothetical birth weight needed to cross the maximum maternal metabolic scope of 2.1 can be calculated as 4.9 kg using the formula in Table S2 of Dunsworth *et al.* [6] (Table 2). This indicates that foetal energy requirements would need to increase from a sustained 383 kcal/d in week 36 to about 750 kcal/d at birth in week 40 (see Fig. 1). In other words, average foetal energy requirements would have to almost double within the four last weeks of gestation. However, to exceed an even higher metabolic ceiling of 2.3 × BMR_NPNL_ and 2.5 × BMR_NPNL_, respectively, the foetal energy requirements would need to increase to about 1050 kcal/d and 1350 kcal/d. This corresponds to almost a tripling or a quadrupling, respectively, of the foetal energy requirements by week 36. At the same time, it would require an extraordinarily high average birth weight of 5.9 kg and 6.9 kg, respectively (Table 2).

Such a steep exponential growth curve is also challenged by a recent analysis taking offspring energy requirement immediately after birth into account, which suggests a linear rather than exponential increase, both immediately before and after birth [10] (see also Fig. 1).

### Limitations of the present study

A potential limitation of our analysis is that some parameters had to be estimated. Accordingly, the lowest increase in sustained metabolic scope during pregnancy among studies performed in socioeconomically stronger countries is shown by the study of Butte *et al.* [46]. This might, however, be due to our potentially imprecise substitution of non-pregnant, non-lactating BMR and TEE, which Butte *et al.* [46] did not measure, by the weighted means of these values from studies performed in economically stronger countries. In fact, different patterns of BMR increase were observed during pregnancy [37], and changes in BMR during pregnancy may vary significantly between women [26]. At the same time, this makes the low increase in sustained metabolic scope during pregnancy plausible that we inferred for Butte *et al.* [46]. Moreover, the high metabolic scope before pregnancy reported by both Dufour *et al.* [47] and Panter-Brick [44] (Fig. 3) could indicate either underestimation of the non-pregnant, non-lactating BMR, or, conversely, overestimation of the non-pregnant, non-lactating TEE. While the high metabolic scopes observed in both studies before pregnancy are unlikely due to the specific measurement method used, a potential source of error might be the age-specific predictive equation of the FAO [60] to estimate BMR, whose accuracy has been questioned [74]. The equation may potentially overestimate non-pregnant, non-lactating BMR, but a lower estimated non-pregnant, non-lactating BMR would consequently lead to an increase of metabolic scope in the studies of Panter-Brick [44] and Dufour *et al.* [47]. Nevertheless, the women in both studies show higher non-pregnant, non-lactating activity energy expenditure compared to the women analysed in other studies. Therefore, they most likely engaged in physically challenging work before pregnancy. Also, activity levels may be reduced during pregnancy. However, it has been suggested that women in socioeconomically weaker countries are unable, voluntarily or otherwise, to reduce their physical activity during pregnancy and lactation [26]. Therefore, we suggest that the high non-pregnant, non-lactating metabolic scopes reported by Panter-Brick [44] and Dufour *et al.* [47] are plausible, which also applies to their relatively high metabolic scopes during pregnancy that only increase slightly until the end of the third trimester compared other studies. Conversely, a low physical activity before and during pregnancy compared to the women of other studies performed in socioeconomically stronger countries was observed by de Groot *et al.* [45] in the Netherlands (Fig. 3). Due to the low physical activity levels, TEE was also lower, which consequently lowered the sustained metabolic scope. Although it is remarkable that the two studies using room calorimetry [45, 46] showed lower sustained metabolic scopes during pregnancy, especially towards the end of the third trimester, the exclusion of these two studies would increase the percentage of studies in which the sustained metabolic rate exceeds the suggested metabolic ceiling of 2.1 × BMR_NPNL_ from 33.3% to 40.0%.

## CONCLUSIONS AND IMPLICATIONS

The Energetics of Gestation and Growth (EGG) hypothesis posits that maternal energy requirements steeply increase during pregnancy and approach a plateau in the third trimester close to 2.1 × BMR of the non-pregnant, non-lactating condition, and that labour starts when the exponentially growing energetic requirements of the foetus cross the maximum sustained maternal metabolic scope [6].

The present meta-analysis of the 12 studies that measured TEE during pregnancy demonstrated, however, (i) considerable variation in the observed sustained maternal metabolic scope. (ii) On average, pregnancy was found to be less energetically costly than suggested previously, and (iii) in the great majority of the studies maternal metabolic scope did not plateau in the third trimester, implying a pattern that is not suggestive of a metabolic ceiling being approached by the maternal energy requirements during pregnancy. Moreover, (iv) a large percentage of the studies significantly exceeded the presumed metabolic ceiling of the EGG hypothesis of about 2.1 × BMR_NPNL_. (v) Because the EGG hypothesis posits that labour is only triggered when the energetic requirements of the foetus surpass the maximum sustained metabolic capacity of the pregnant woman, a metabolic ceiling during pregnancy exceeding 2.1 × BMR_NPNL_ would imply an unrealistically high mean birth weight of > 4.9 kg. Conversely, (vi) the remarkably high variation in energy expenditure of the pregnant women strongly contrasts with the relatively low variation in gestation length. If birth timing were in fact dependent on energy expenditure during pregnancy, gestation length would likely be equally variable, which questions the conceptual basis of a metabolic ceiling in determining the onset of labour.

A metabolic trigger of human birth has also been challenged by a study of obstetric selection pressures in early hominid fossils using FEA birth simulations, demonstrating that cephalopelvic constraints were similarly high in australopithecines as in modern humans [5]. This implies that secondary altriciality evolved in the absence of an increased foetal size and thus with foetal energy requirements comparable to those of extant great apes [5]. It is, therefore, not surprising that in the past years, multiple redundant pathways have been discovered in humans that complement the hypothalamic–pituitary–adrenal axis and act synergistically to set the human parturition clock, including foetal membrane ageing, telomere senescence, decidual senescence, progesterone withdrawal and diverse inflammatory mediators [30].

Independent of the question of birth timing and the EGG hypothesis, it remains unclear from an evolutionary point of view why maternal metabolic capacity would be limited given the potential negative consequences. Further, the significantly higher costs of lactation make it more plausible that this activity might be pushing humans closer to the limit of their energetic capacity, wherever such a threshold might lie. Yet, it is well known that even human lactation can well be supported through increased dietary intake rather than substantial mobilization of adipose tissue [3, 26, 44, 46, 54, 75]. The ability of humans to continually engage in endurance activities while pregnant and while lactating, with physically active women tending to have higher milk production [76], is further evidence that humans are not necessarily at their energetic limit during pregnancy and lactation. As such, further study is needed to understand maternal metabolism and the proximate mechanisms determining sustained metabolic scope itself and whether its interpretation as a ‘metabolic ceiling’ is truly warranted.
